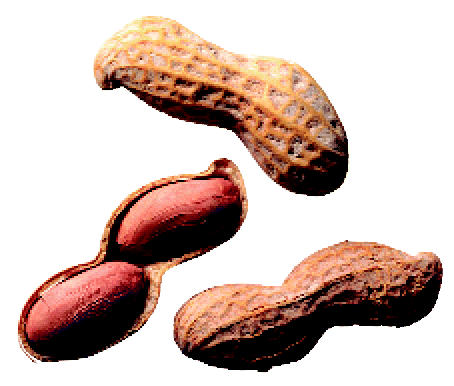# The Beat

**Published:** 2004-12

**Authors:** Erin E. Dooley

## Dodging the Bullet

Studies in the late 1990s showed that lead bullets were contaminating soil and groundwater near shooting ranges. Alternative “green” bullets made of tungsten were developed and first distributed to select Army facilities in 1999. Now researchers at the Stevens Institute of Technology have found that tungsten and its alloys dissolve in water and soil at rates that exceed lead. And tungsten, once thought to be a benign substance, is being investigated by the CDC as possibly contributing to a cluster of leukemia cases in Fallon, Nevada, where the mineral occurs naturally. Military bases are examining possible ways to capture the not-so-green bullets or prevent their leaching. Meanwhile, tungsten has been nominated for toxicity study by the National Toxicology Program.

## The Worm Turns in Cambodia

Cambodia has successfully treated 75% of its nearly 3 million school-aged children against intestinal worms six years ahead of a World Health Organization goal for global parasite control. Cambodia is the first country to reach this international goal. The inexpensive treatment (2¢ per pill) can be administered by teachers in classrooms, as is being done in Cambodia.

Worldwide, intestinal worms affect at least 2 billion people. Children affected with intestinal worms weigh less than healthy children and are more prone to anemia. Left untreated, infection with intestinal worms can cause irreversible organ damage and impaired intellectual development. Once treated, however, affected children’s short- and long-term memory, reasoning capacity, and reading comprehension all improve dramatically, and school absenteeism drops as much as 25%.

## Wave of Fish Advisories

New figures released by the U.S. EPA show that 35% of lake acreage in the United States and 24% of river miles contain enough pollution to warrant consumption advisories for fish caught in those waters. The advisories cover some 40 different substances; 98% of them involve PCBs, chlordane, dioxins, DDT, or mercury. Though the number of advisories rose from 2,814 in 2002 to 3,094 in 2003, EPA administrator Mike Leavitt attributed that increase to more monitoring rather than an increase in pollution. Environmental advocates say these latest findings point up the need to more strictly regulate coal-fired power plants, one of the primary sources of mercury.

## Malawi Bans Methyl Bromide

Although the Montréal Protocol stipulates that methyl bromide need not be banned in developing countries until 2015, protocol party Malawi is working to phase out use of the ozone-depleting pesticide by the end of 2004. Imports of the pesticide after 31 December 2004 are to be impounded.

Malawi is the second largest user of the pesticide in Africa, and tobacco is one of the country’s principal sources of foreign cash flow. The ban will make the country the first in its region to phase out nonessential uses of the pesticide. The Malawian Agricultural Research and Extension Trust is working to raise awareness among the country’s farmers about the hazards of using the chemical and about alternatives to using it, which include soil-less culture of tobacco plants and use of more benign chemicals such as dazomet.

## Plutonium Accumulating in Japanese Bay

Fifty years ago, the United States performed tests of nuclear weapons in the Marshall Islands, an island group almost halfway between Hawaii and Tokyo. Now radioactive plutonium particles that match the fallout from those blasts have been found in Japan’s Sagami Bay by researchers at the Japanese National Institute of Radiological Science. This is the first time such particles have been found in Japanese waters.

The researchers believe they pose no environmental risk. They plan to study other shorelines in Japan to determine how the particles traveled—useful information in the event of a nuclear emergency. At present researchers believe the particles were carried by the ocean currents.

## Protecting Peanuts from Aflatoxins

The U.S. Agricultural Research Service has received EPA approval for the first biological pesticide to protect peanut crops against toxic *Aspergillus* mold strains that produce aflatoxins. Consumption of grains and nuts contaminated with aflatoxins has been linked with liver cancer and hepatitis in humans. Afla-Guard, as the new treatment is known, is from a nontoxigenic strain of *flavus*. It is applied beneath the plant canopy, where it competes against its aflatoxin-producing cousins, which generally colonize plants that are stressed by drought conditions. Afla-Guard also works on peanuts that are stored in warehouses. In field trials, the treatment reduced aflatoxin contamination by 70–90% after the first application, and even more with subsequent applications.

## Figures and Tables

**Figure:**
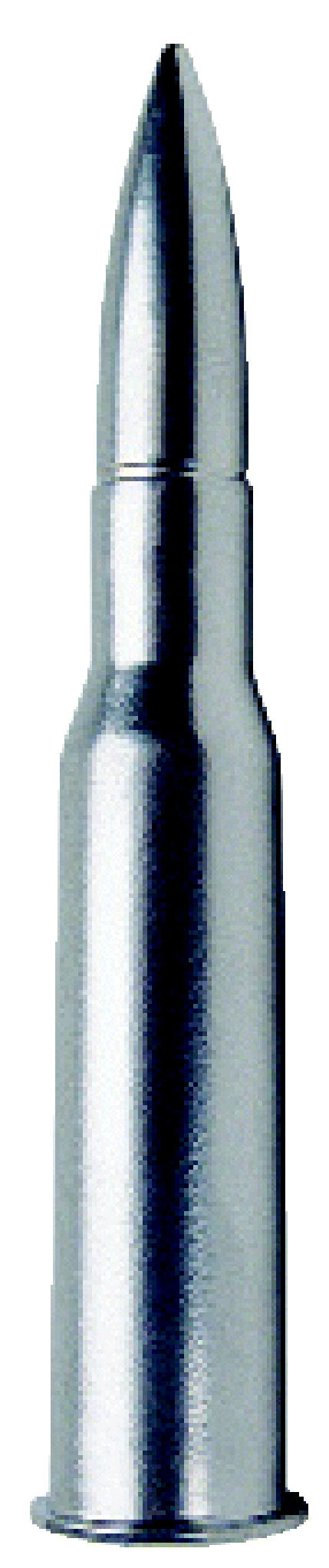


**Figure:**
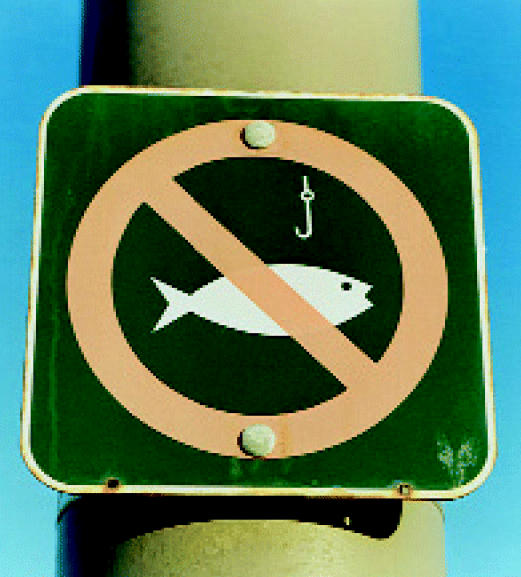


**Figure:**
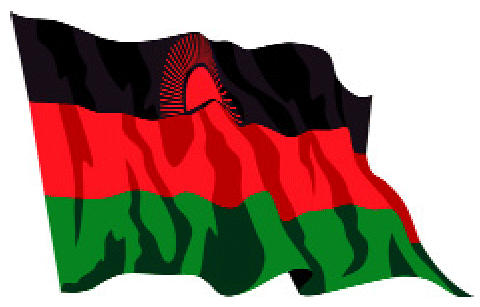


**Figure:**